# Fish Reproduction Is Disrupted upon Lifelong Exposure to Environmental PAHs Fractions Revealing Different Modes of Action

**DOI:** 10.3390/toxics4040026

**Published:** 2016-10-28

**Authors:** Caroline Vignet, Thibaut Larcher, Blandine Davail, Lucette Joassard, Karyn Le Menach, Tiphaine Guionnet, Laura Lyphout, Mireille Ledevin, Manon Goubeau, Hélène Budzinski, Marie-Laure Bégout, Xavier Cousin

**Affiliations:** 1 Ifremer, Ecotoxicology Laboratory, Place Gaby Coll, F-17137 L’Houmeau, France; vignet.caroline@gmail.com (C.V.); llyphout@gmail.com (L.L.); manon.goubeau@gmail.com (M.G.); 2 INRA UMR703, APEX, Oniris, F-44307 Nantes, France; thibaut.larcher@oniris-nantes.fr (T.L.); mireille.ledevin@oniris-nantes.fr (M.L.); 3 Oniris, École Nationale vétérinaire, Agro-Alimentaire et de L’alimentation Nantes-Atlantique, LUNAM Université, F-44307 Nantes, France; 4 Department of Science and Technology, University of Bordeaux 1, EPOC, UMR CNRS 5805, F-33405 Talence, France; b.davail@epoc.u-bordeaux1.fr (B.D.); k.lemenach@epoc.u-bordeaux1.fr (K.L.M.); h.budzinski@epoc.u-bordeaux1.fr (H.B.); 5 Ifremer, Fisheries Laboratory, Place Gaby Coll, F-17137 L’Houmeau, France; lJoassar@ifremer.fr (L.J.); tiphene9@hotmail.com (T.G.); mlbegout@ifremer.fr (M.-L.B.); 6 INRA LPGP, Campus de Beaulieu, F-35042 Rennes, France; 7 Ifremer, Laboratoire Adaptation et Adaptabilité des Animaux et des Systèmes, UMR MARBEC, Route de Maguelone, F-34250 Palavas les Flots, France

**Keywords:** polycyclic aromatic hydrocarbons, zebrafish, spawning success, fertilization, gonad differentiation, molecular mechanisms

## Abstract

Polycyclic aromatic hydrocarbons (PAHs) constitute a large family of organic pollutants emitted in the environment as complex mixtures, the compositions of which depend on origin. Among a wide range of physiological defects, PAHs are suspected to be involved in disruption of reproduction. In an aquatic environment, the trophic route is an important source of chronic exposure to PAHs. Here, we performed trophic exposure of zebrafish to three fractions of different origin, one pyrolytic and two petrogenic. Produced diets contained PAHs at environmental concentrations. Reproductive traits were analyzed at individual, tissue and molecular levels. Reproductive success and cumulative eggs number were disrupted after exposure to all three fractions, albeit to various extents depending on the fraction and concentrations. Histological analyses revealed ovary maturation defects after exposure to all three fractions as well as degeneration after exposure to a pyrolytic fraction. In testis, hypoplasia was observed after exposure to petrogenic fractions. Genes expression analysis in gonads has allowed us to establish common pathways such as endocrine disruption or differentiation/maturation defects. Taken altogether, these results indicate that PAHs can indeed disrupt fish reproduction and that different fractions trigger different pathways resulting in different effects.

## 1. Introduction

In ecology, recruitment is defined as the ability of one individual to contribute to the next generation. From birth, this includes a large number of abilities such as to develop, to grow, to meet congeners and to reproduce. Beyond intrinsic characteristics, genetically defined, many external factors can impede recruitment. Among them, xenobiotics could act on virtually all functions or important steps [1].

Among xenobiotics, polycyclic aromatic hydrocarbons (PAHs) form a diverse family of compounds containing at least two aromatic rings [2]. PAHs are increasingly emitted in the environment as a consequence of human activities [3,4]. There are two major types of environmental PAHs, petrogenic and pyrolytic, which enter the environment through different routes. Petrogenic PAHs are abundant in oils and enter the aquatic environment due to harbor activity or as a consequence of oil spills. Pyrolytic PAHs result from the incomplete combustion of organic matter, including fossil fuel; they enter aquatic environments through the deposition of atmospheric emissions directly on water or on the ground followed by soil runoff. Because PAHs are hydrophobic molecules, they are found to be associated with suspended particulate matter in water, such that they tend to accumulate in sediments; consequently, sediments constitute major sinks and can also act as secondary sources for aquatic systems contamination [5]. Monitoring networks have documented PAH concentrations of up to 50 μg·g^−1^ dry weight (dw) in sediment from various affected aquatic ecosystems although the concentrations in highly contaminated areas are more commonly in the 10 μg·g^−1^ range [6,7,8,9,10,11].

Thanks to its biological characteristics, including a short life cycle, the zebrafish makes a particularly good case study for performing longitudinal studies along the life cycle. This applies in the case of reproduction for which it is possible in some months to monitor the gonads’ development and maturation, endocrine regulation and reproductive effort. In addition, the large numbers of tools developed to study zebrafish physiology allows for a refined analysis [12,13].

Reproduction is a key function to any species survival, but, at the same time, it is the target of some xenobiotics. Reproduction disruption may be the results of direct insults such as reproduction hormones modification in the case of endocrine disruptor compounds or may be the indirect consequence of general physiological degradation including growth or behavioral defects [14]. Additional complexity is due to the fact that reproduction is the ultimate step in a long-term process, from germ-cells’ migration in embryos to gametes’ maturation in reproducing adults, and that all these steps can be disrupted throughout life and eventually result in reproduction failure. Complexity is also due to the fact that, in the environment, xenobiotics are present as complex mixtures of compounds which, taken separately, can have pro- or anti-estrogenic properties [15]. PAHs have been shown to interfere with reproduction in fish and other animals, including invertebrates [16,17,18,19,20,21,22,23,24,25]. From a mechanistic point of view, many elements remain unclear; however, PAHs have been shown to produce endocrine disruption in fish, which has been revealed by disruption of circulating steroid hormones level in males and females [16,26,27,28,29,30].

In previous articles we have reported physiological disruptions observed in zebrafish after long-term diet exposure to environmentally relevant mixtures of polycyclic aromatic hydrocarbons (PAHs) and demonstrated disruption of growth, behavior and survival along with an increase in tumorigenesis [31,32,33]. In order to evaluate the reprotoxicity of environmental PAHs mixtures, zebrafish were exposed through diet from the first meal (5-days post fertilization (dpf)) and for the 9 following months, i.e., from larvae to reproducing adults. Three complex fractions of PAHs, representative of environmental situations, were used to spike the food: a pyrolitic (PY) fraction from sediment sampled in the Seine Estuary; and two petrogenic fractions, one from a heavy oil “*Erika*” (HO) and one from a light crude oil “Arabian light” (LO). We analyzed the consequences of this exposure on reproduction evaluated at functional, organ and molecular levels.

## 2. Material and Methods

### 2.1. PAH-Contaminated Diet Preparation

Three aromatic fractions were used for exposures: (i) A pyrolytic fraction (PY) extracted from sediments collected in a polluted site of the Seine Estuary (Oissel, France) and (ii) two petrogenic fractions obtained from Erika fuel (heavy oil; HO) and Arabian Light crude oil (LO). PAHs extractions were performed as previously described [7]. Zebrafish TU strain (ZFIN ID: 76 ZDB-GENO-990623-3) was used and larvae were exposed from their first meal (at 5 dpf) onward using spiked food pellets [33]. Three concentrations, 0.3X, 1X and 3X were used with the 1X concentration corresponding to the 16 PAHs used as indicators by the US Environmental Protection Agency (US-EPA) at 5 μg·g^−1^ dw food, representative of the concentrations found in mollusks in the Seine Estuary. Although it does not give an accurate representation of the fractions’ compositions, this subset of PAHs has been used for comparison with concentrations found in the environment (see in Table S1 a detailed description of actual fractions compositions). Diets are named after the origin of the fraction and its concentration: the 1X pyrolytic fraction diet will be named PY-1X. For each exposure, a fourth control treatment was included corresponding to the plain food treated as spiked-food with dichloromethane which was used as carrier solvent for PAHs spiking. Because the fraction extracted from the original sample is a mixture of PAHs, the term mixture will also be used more or less synonymously with fraction.

### 2.2. Reproduction Monitoring

Fish were reared in triplicates as batches of 25–30 individuals in 10 L tanks (see [33] for further details). Onset and spawning effort were determined by placing two spawning boxes (AquaSchwartz, Göttingen, Germany) at 17:00 on the bottom of each 10 L tank from 4 to 6 months post fertilization (mpf) in PY fish, from 4 to 7 mpf in HO and from 5 to 9 in LO fish. In total, 19 to 87 spawning assays (solicitation) were performed depending on fraction and concentration (Table 1). Further monitoring of reproduction effort was performed with cross-pairing for LO and HO (without HO-3X) using one exposed fish and one control fish pairwise mated overnight in a spawning box (AquaSchwartz, Germany) outside of the tank. In both cases (group and pairwise mating), spawning occurs the following morning progressively after a few minutes of light illumination. One hour later, eggs were collected, cleaned, sorted (into fertilized or none fertilized), and counted. The monitoring allowed us to assess spawning success (ratio of number of spawns obtained/number of assays), egg production (cumulative number of fertilized egg over observation time further normalized by the number of assays and the number of females in the tank) and spawn fertilization rate.

### 2.3. Histological Analysis

Fish were sampled at 9 mpf for all diets except for HO which were sampled at 7 mpf because of high mortality in HO-3X condition. Control fish were systematically sampled in parallel. Fish were euthanized with a lethal dose of benzocaine (250 μg·L^−1^ of a 10% solution in 100% ethanol; Sigma-Aldrich, Lyon, France) and fixed in 4% buffered formalin after ventral incision of the abdomen for larger individuals [34]. After 24 h of fixation, fins, most scales and the caudal peduncle were carefully removed and samples were dehydrated in graded ethanol solutions and embedded in paraffin. Step sectioning was performed as already published [35]. Serial sagittal step sections were cut from the left side of the fish. Four step sections from each adult fish were mounted on glass slides, one from the eye anterior chamber level, one from the eye posterior chamber level, one just medial to the eye, and one at the midline. Sections were routinely stained with haematoxylin-eosin-saffron (HES). A first histological analysis was performed to qualify structure and morphology on the gonads and also to identify lesions. The second step consisted of follicle staging (functional characterization). All oocytes present on mid-level section and on sections medial to the eye were counted along with identification of their morphological stage (F1 to F4) using middle magnification [36]. Individual frequencies of each type of follicles were calculated using ratio to the total ovarian area.

### 2.4. qPCR Analysis

Ovaries and testis were sampled on 9mpf animals from each fraction and concentration except HO-3X (*N* = 5 per case) euthanized as described above and immediately frozen in liquid nitrogen and stored at −80 °C until used. Total RNAs were extracted using Trizol Reagent (Invitrogen, Carlsbad, CA, USA) following the manufacturer’s instructions. Total RNA concentration was quantified by spectrophotometry at 260 nm. Furthermore, purity of RNAs was verified by measuring the A260/A230 nm and A260/A280 nm ratios. To remove the possibility of genomic DNA contamination, RNA sample was digested by RNase-free DNase I (Promega, Madison, WI, USA) and then purified. First-strand cDNA was synthetized from a total of 1 µg RNA using reaction mix including 500 ng of oligo(dT)15, 250 ng of random hexamerprimers (Promega, Madison, WI, USA) using M-MLV Reverse Transcriptase (Promega, Madison, WI, USA) following manufacturer’s instructions. Reaction was incubated for 1 h at 42 °C and inactivated by heating for 15 min at 70 °C and cDNA stored at −20 °C until a real-time PCR analysis. PCR primers were designed with Primer 3 software. Real-time PCR reactions were performed using Fast SYBR Green Master Mix 5X (ThermoFisher, Illkirch, France) and in a StepOnePlus instrument (ThermoFisher, Illkirch, France) following the manufacturer’s instructions. Gene’s expressionwere quantified from the threshold cycle (CT) number and normalized to four housekeeping genes (*eef1*, *βactin*, *g6pd* and *gapdh*). Gene expressions were analyzed in females (*esr1*, *esr2a*, *esr2b*, *cyp19a1a*) and males (*amh*, *spag8*, *cyp17a1*, *hsd11b3*) using REST-2009 software (Qiagen, Dusseldorf ,Germany).

### 2.5. Statistical Analysis

Within each fraction (PY, LO, HO), fertilization rate frequencies were calculated per classes (20% wide) and Chi2 analysis performed to compare eggs fertilization rate as well as spawning success between concentrations using Prism 6.0 (Graphpad, La Jolla, CA, USA ).

Comparing fertilization rates in cross-pairings was performed using non-parametric test, Kruskal-Wallis, whereas GLMs were calculated to compare egg production within each fraction (PY, HO or LO) with concentration as fixed factors (Control, 0.3X, 1X and 3X). For PY ovary morphology, surface of degenerating areas expressed as percentage of total ovarian surface were compared between concentrations (Control, 0.3X, 1X and 3X) using ANOVA. For ovary quality evaluation within each fraction, concentration and follicles stages (F1 to F4) were used as a fixed factor and fish as random factor. Interaction between concentrations and stages was also analyzed. Post hoc tests were performed with Newman-Keuls in all cases. These statistical analyses were performed with Statistica 9.0 (Statsoft, Tulsa, OK, USA) software.

All statistical analyses were carried out at a 95% level of significance and only the fixed factor and the interaction between them are presented in the text. The results reported in text and all figures are means ± SEM.

## 3. Results

This work is part of a larger program that included analysis of modification of several physiological variables following exposure to the same diets. Chemical analysis of fractions, diets and metabolites have been presented previously [33]. Briefly, PY fraction was characterized by a high proportion of heavy PAHs (82%) and almost no methylated derivatives (<5%). HO fraction contained moderate levels of heavy PAHs (31%) and methylated derivatives (45%). LO fraction contained a very low level of heavy PAHs (6%) and a high level of methylated ones (59%). As mentioned above, proportions of light, heavy and methylated PAHs differ notably between fractions and spiked diets. Total PAHs concentrations based on 22 parents HAPs (including the 16 EPA PAHs) and 8 methylated PAHs are presented in detail in Table S1. Briefly, for PY, HO and LO respectively PAHs parents’ concentrations in 1X diets were 5.5 ± 1.4, 2.6 ± 0.2 and 2.7 ± 0.2 µg·g^−1^ dw and 0.3 ± 0.1, 2.1 ± 0.2 and 4.0 ± 0.3 µg·g^−1^ dw for methylnaphthalenes and methylphenanthrenes. This resulted in a similar total concentration of quantified PAHs (included methylated) in the three 1X diets: 5.8 ± 1.4, 4.7 ± 0.4 and 6.7 ± 0.3 µg·g^−1^ dw for PY, HO and LO respectively. Since PAHs is a family of hundreds of compounds, it is not possible to quantify all individual PAHs and therefore we acknowledge that the above concentrations only partially describe the fractions used. Quantification of hydroxylated metabolites in dietary exposed 15 dpf-larvae confirmed an effective exposure of fish, and results were consistent with diet composition [33].

### 3.1. Reproduction Monitoring

Experimental groups showed varied sex-ratio with females to males (F/M) ratio ranging from 3.32 (PY Control) to 0.51 (HO-1X) while the overall mean ratio was 1.15. When considering only Control conditions, HO-Control had a F/M ratio of 0.54 to PY-Control (F/M = 3.32) or LO-Control (F/M = 1.19). The varying sex ratio arose from the initial creation of the replicate at embryonic stage and the fact that reared replicate composition was not changed over time.

In a first step, group spawns were monitored directly in the rearing tanks starting at 4 mpf for PY and HO fish and at 5 mpf for LO fish. Results are summarized in Table 1. A significant number of spawns were obtained for all diets but 3X concentration for all three fractions with only one spawn was obtained for PY-3X over 87 assays and none for HO-3X and LO-3X. The spawning success rate was significantly reduced after exposure to all three PAHs fractions (Chi2; *p* < 0.001). In more detail, this rate was reduced for all fractions and all concentrations compared to their respective control (*p* < 0.05) except for HO-0.3X (*p* = 0.81).

**Figure 1:**
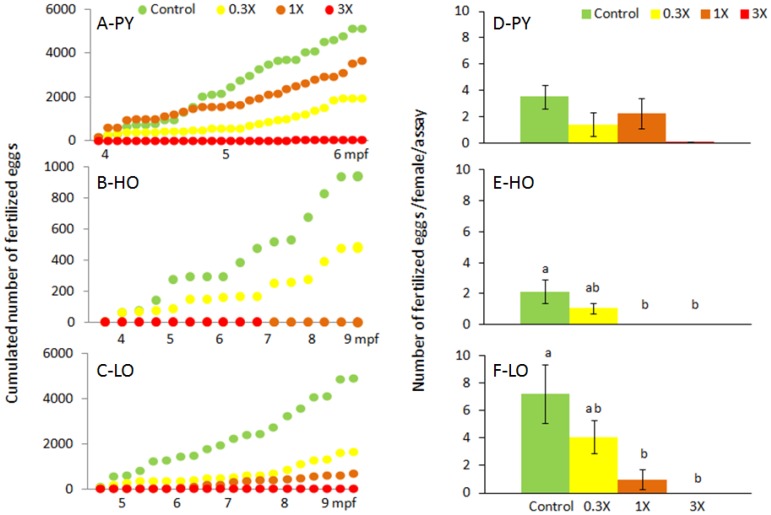
Monitoring of group-spawning. Cumulated number of fertilized eggs over-time (**A**–**C**) and number of fertilized eggs normalized to the number of spawning assays and the number of females in each tank (**D**–**F**) are presented for fish exposed through diet to PY (**A**,**D**), HO (**B**,**E**) and LO (**C**,**F**). In (**D**–**F**) mean ± SEM. Different letters indicate significant difference (*p* < 0.05). Note that in (**A**–**C**), *x*- and *y*-axes scales are different between conditions.

The cumulative number of fertilized eggs was monitored over time (Figure 1A–C).

The number of fertilized eggs spawned per female and assay was significantly reduced for fish exposed to petrogenic diets for 1X and 3X concentrations (Figure 1D–F). The 0.3X was intermediate. In the case of PY-3X, a trend was only obtained (*p* = 0.078) using the GLM analysis despite the fact that only one spawn was obtained with a hundred-fold reduction of the number of fertilized eggs per female per spawn.

**Figure 2:**
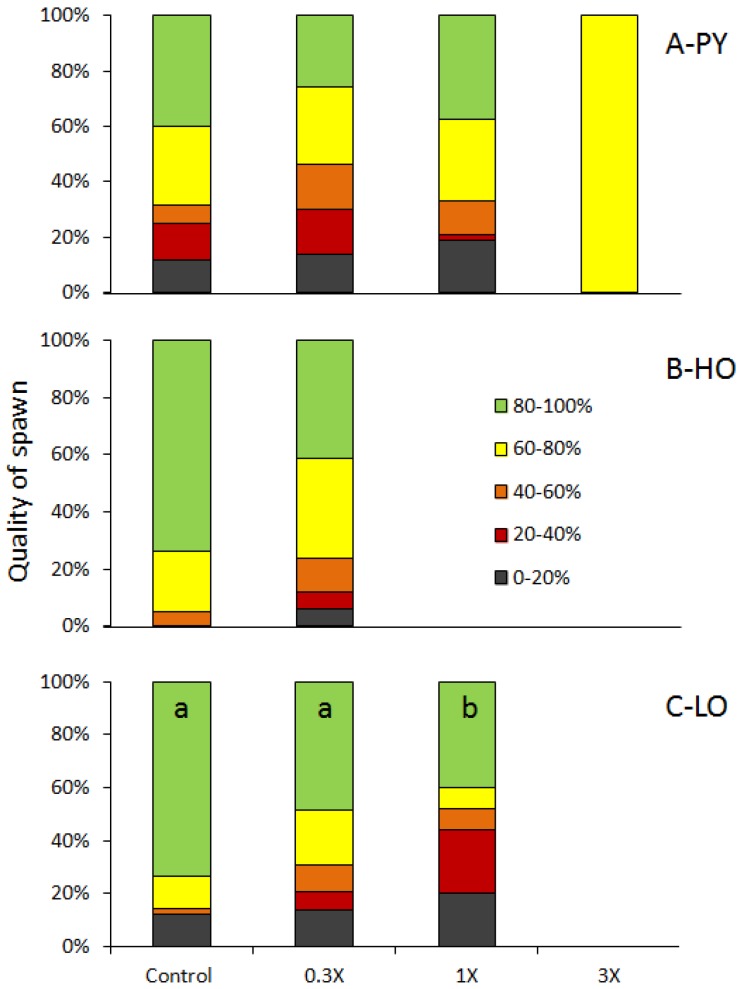
Spawns quality. Group-spawns obtained from adults exposed to pyrolitic (PY) (**A**), heavy oil (HO) (**B**) and light oil (LO) (**C**) diets were classified according to their fertilization rates (see insert in (**B**)). In (**C**), different letters indicate significant difference (*p* < 0.05).

Spawns mean fertilization rates ranged from 66.0% to 77.1% for PY fraction, from 81.8 to 88.1 for HO fraction and from 51.3 to 78.3 for LO fraction with a trend to follow a dose-effect decrease for this latter fraction. When classified according to their fertilization rates in 20% wide classes (Figure 2), this trend was confirmed (overall Chi2 = 18.48; *p* = 0.02). LO-0.3X spawns tended to have a lower quality compared to Control (Chi2 = 7.17; *p* = 0.13) while LO-1X spawn quality was significantly reduced compared to Control (Chi2 = 14.60; *p* = 0.006). GLM analysis indicated that these trends and differences were due to the reduction of the spawn number in class 80%–100% for LO-0.3X and LO-1X (*p* = 0.09 and *p* = 0.05 respectively).

**Figure 3:**
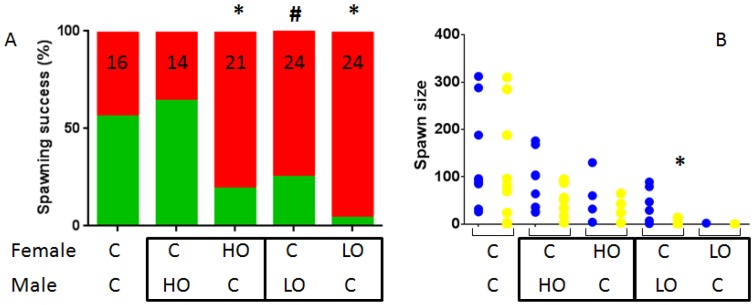
Qualification of Control/contaminated spawns. Spawning success of pairwise mating using fish from different conditions. Successful spawns are indicated in green while unsuccessful ones are in red. The number of assays is indicated in each bar (**A**); Number of eggs (blue) and fertilized eggs (yellow) of each successful spawn (**B**). * indicates success differing from Control homogenous pairs (*p* < 0.05) and ^#^ trend to a difference (*p* < 0.1).

In order to evaluate if reproduction disruption could be ascribed to a particular sex and so gain insight into mechanisms, we performed crosses between exposed and non-exposed fish. This was performed using HO-1X and LO-3X fish. Contrary to what was observed with homogenous fish, we obtained spawns for all crosses involving contaminated fish albeit with various success (Figure 3). Spawning success for female Control/male Control crosses was obtained in 9 assays out of 16 (56.3%). Spawning success for female Control/male HO-1X crosses was not different (64.3%; *p* = 0.72). In contrast, in female HO-1X/male Control crosses, the success was significantly reduced compared to Control (19.0%; *p* < 0.05). In the case of LO-3X crosses, the situation was different with a trend to decrease for female Control/male LO-3X (25.0%; *p* = 0.09) and a very significant decrease for the opposite cross female LO-3X/male Control with only one spawn obtained out of 24 assays (4.2%; *p* < 0.001). When further qualifying the obtained spawns, only one spawn of the female LO-3X/male Control cross was excluded from the analysis; the only significant difference obtained was the number of fertilized eggs obtained in the female Control/male LO-3X crosses which was significantly reduced (KW = 14.95; *p* = 0.02 and post-hoc test *p* = 0.04), resulting in a fertilization rate of 7% which is approximately tenfold lower compared to other conditions (60%–84%).

### 3.2. Histological Analysis

**Figure 4:**
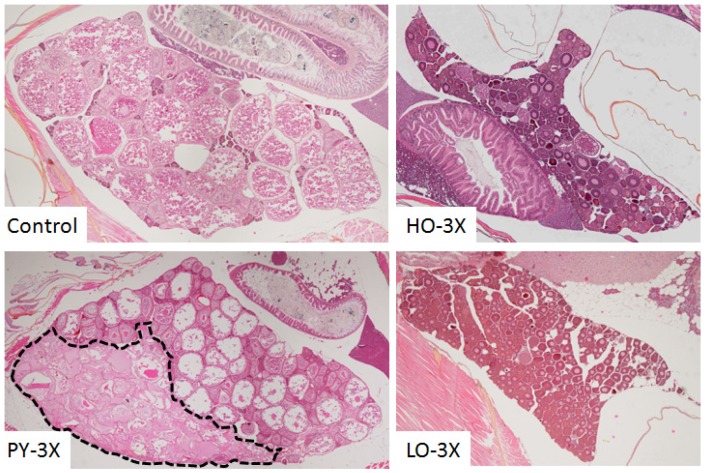
Ovary morphology. Representative pictures of ovaries sampled from Control, PY-3X, HO-3X and LO-3X females. The dotted line delimits disrupted area in PY-3X ovary.

Histological analyses were performed to assess the ovaries’ structure, morphology and functionality. The ovaries’ overall structure was affected in female exposed but these disruptions differed depending on the fraction (Figure 4).

**Figure 5:**
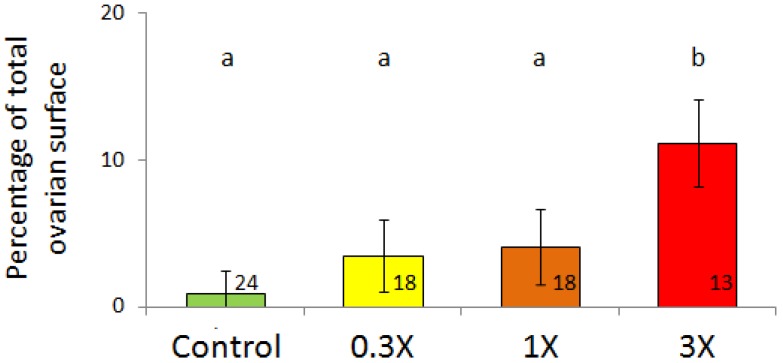
Surface of degenerating areas in PY fish ovaries (% of total ovarian surface). Mean ± SEM, different letters indicate significant difference (*p* < 0.05). The number of individuals analyzed is indicated in each bar.

Ovaries from control females presented an even distribution of follicles at all stages of maturation. All stages of follicles were also found in the ovary from females exposed to PY-3X diet which presented large areas of disrupted morphology which did not correspond to atretic follicles but rather degenerating areas. The surface percentage of these degenerating areas increased with PY concentration (Figure 5; *F* = 4.81; *p* = 0.007). This kind of degenerating area was not found in ovaries from HO-3X and LO-3X fish.

**Figure 6:**
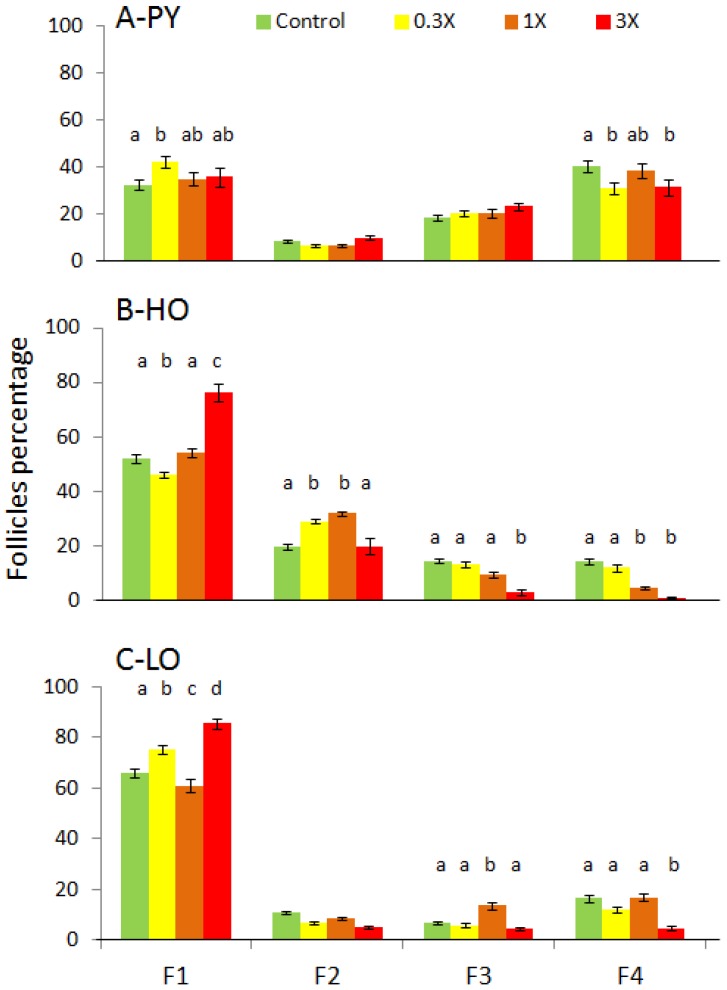
Follicles distribution according to their stage. Follicles were classified according to their maturation: pre-vitellogenic stages (F1 and F2); post-vitellogenic stage (F3) and mature follicles (F4). Percentage of follicles in each class, mean ± SEM. Different letters indicate significant difference compared to the relative control (*p* < 0.05).

Ovaries from females exposed to petrogenic fractions presented a significantly reduced number of maturing follicles (Figure 4). To quantify this disruption, follicles were counted according to their stage (Figure 6). In PY ovaries at 9 mpf, a slight increase in the proportion of F1 was observed in PY-0.3X ovaries. In contrast, a slight reduction of F4 was observed for PY-0.3X and PY-3X fish. More important disruptions were observed in HO and LO females. A significant increase of F1 was observed in HO-3X and LO-3X and to a lesser extent in LO-0.3X. In contrast, a slight decrease of F1 was observed in HO-0.3X and LO-1X. The percentage of some intermediate follicles was increased, F2 for HO-0.3X and HO-1X as well as F3 for LO-1X or decreased F3 for HO-1X. The case of F4 is clearer with a significant decrease of mature follicles in ovaries from HO-1X, HO-3X and LO-3X.

**Figure 7:**
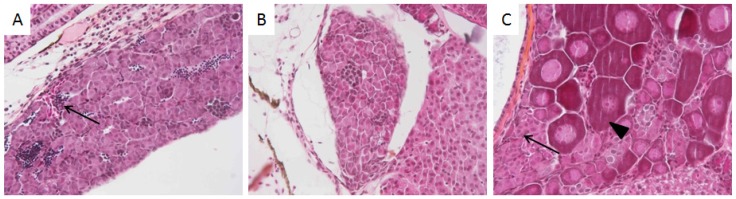
Testis functional disruptions. Testicular hypoplasia revealed by the low number of spermatozoa (**A**) or their absence (**B**). Ovotestis observed in HO-3X with coexistence of immature follicles (arrowhead) and spermatogonia (arrow) (**C**).

Testis structure and morphology were also analyzed at the same age. Testicular hypoplasia (Figure 7A,B) has been observed in males exposed to HO-1X (*n* = 3; 6.4% of analysed males), in HO-3X (*n* = 9; 42.9%) as well as in LO-3X (*n* = 2; 7.4%). In addition to these functional disruptions, some seminoma have been identified in 1X or 3X concentrations in all fractions while none has been found in control fish. These very invasive tumors were described in more detail in Larcher et al. [31]. Finally, one ovotestis has been identified in HO-3X treatment (Figure 7C).

### 3.3. Genes Expression Analysis

Expression of genes encoding estrogens receptors (*esr1*, *esr2a* and *esr2b*) and ovarian aromatase (*cyp19a1a*) was assessed in ovaries sampled at 9 mpf (Table 2). Regarding estrogens receptors, the *esr1* gene is up-regulated in ovaries of PY-1X (*p* < 0.05) and HO-0.3X (*p* < 0.05) while *esr2a* is up-regulated in HO-0.3X (*p* < 0.01) and HO-1X (*p* < 0.05). In contrast, *esr2b* is down-regulated in LO-1X (*p* < 0.05) and just above significance threshold for LO-3X (*p* = 0.053). Ovarian aromatase is also up-regulated in ovaries of PY-1X (*p* < 0.05) and HO-0.3X (*p* < 0.05) but down-regulated in ovaries of LO-3X.

In testis, genes expression has been monitored for two genes involved in the steroidogenesis pathway (*cyp17a1* and *hsd11b3*) and two genes are an indicator of spermatogenesis (*amh* and *spag8*; Table 3). In testis of exposed fish, *spag8* is down-regulated in PY-0.3X (*p* < 0.01) and PY-1X (*p* < 0.01) while *cyp17a1* is down-regulated in PY-0.3X (*p* < 0.01), HO-0.3X (*p* < 0.01) and H0-1X (*p* < 0.01). In contrast, *cyp17a1* is up-regulated in L0-1X (*p* < 0.01) as well as *amh* in L0-0.3X (*p* < 0.05) and L0-1X (*p* < 0.01) and just above the significance threshold for LO-3X (*p* = 0.065).

Fold change is indicated relative to ovaries sampled in Control females. The 95% confidence interval provided by REST software is provided (95% CI). Values in bold are significantly different compared to expression in Control fish.

Fold change is indicated relative to testis sampled in Control males. The 95% confidence interval provided by REST software is provided (95% CI). Values in bold are significantly different compared to expression in Control fish.

## 4. Discussion

### 4.1. Reproductive Disruptions

Several reproductive traits monitored in this study were disrupted upon lifelong exposure to PAHs fractions all contributing to a reduction in reproduction success. Some differences were observed between fractions and will be specifically discussed later on. The most affected trait is the spawning success evaluated as the number of spawns collected over a period of time. For all fractions, a complete failure of spawning was observed for the 3X concentration (except PY-3X; 1 spawn out of 87 attempts). In addition, zero spawn was obtained for HO-1X. Otherwise, a general trend towards a dose-effect reduction in spawning success could be observed.

Tank replicates were established before hatching and remained unchanged during the whole experiment in order not to perturb group composition in each rearing tank. This was a methodological choice based on the fact that visual and olfactive cues have been shown to be important for reproduction [37]. Because sex is plastic in zebrafish [38], this could result in groups with biased sex-ratio which was indeed observed in our experimental groups with a females to males (F/M) ratio ranging from 3.32 (PY Control) to 0.51 (HO-1X) while the overall mean ratio was 1.15. These differences in sex-ratio may explain some differences in terms of spawning success between diets. When considering only Control conditions, HO-Control with a F/M ratio of 0.54 had a 51% spawning success compared to PY-Control (F/M = 3.32; Success = 69%) or LO-Control (F/M = 1.19; Success = 77%) which suggests that if a low ratio could result in a lower spawning success there is no obvious correlation. Considering relative sex-ratio and spawning success, this may have only lead to an underestimation of spawning success for L0-0.3X condition. To further characterize reproduction traits, spawn’s size normalized per number of females and successful assays in each tank revealed an additional disruption for LO-1X, while spawn’s size from HO- and LO-0.3X were intermediate between Control and 1X conditions. Finally, fertilization rates decreased significantly for LO-1X spawns. Altogether this resulted in a dramatic decrease in reproductive output for exposed fish, as evaluated by the cumulative number of produced fertilized eggs. The analysis of all those traits are rarely considered altogether, especially in such kinds of ecotoxicological studies. Therefore, results will be globally discussed in the light of relevant studies which have, however, mostly been performed using individual PAHs. Indeed, exposures to individual PAHs have been shown to reduce the number of eggs in several fish species [19,39,40,41,42] while an exposure to benzo[a]pyrene produced a decreased fertilization rate in mummichog [43]. In contrast, an exposure of Japanese medaka to phenanthrene produced no modification of the daily number of eggs spawned and fertilization rate did not change, with the exception of one intermediate concentration [17].

The paucity of information on reproduction functionality is puzzling owing the number of studies reporting the disruption of biomarkers related to reproduction after exposure to PAHs. If some studies have reported no gonadal disruptions [17,43], some experimental exposure to PAHs as well as analysis of fish collected in the field in PAH-contaminated areas revealed disruption of gonadal structure or reproduction-related biomarkers, which could help to understand the presently observed reproductive defects. For example, some studies showed that fish exposure to PAHs results in gonadal structure disruption indicative of maturation defects in females [39,40,44] and in males [44,45]. Our results are in agreement with these articles, revealing a decrease in ovary maturation as shown by a decrease in mature follicles (F4) proportion in female exposed to 3X concentration for all fractions and post-vitellogenic follicles (F3) for HO-3X females. In the case of PY females, we have also identified degeneration areas in ovaries, the relative surface of which increased with PY concentration. In the case of males, the observed defects (hypoplasia in a small proportion of LO-3X and HO-1X and in a high proportion (42%) of HO-3X males) have already been mentioned in cod after exposure to produced water [44]. Further, one ovo-testis was also found in one individual morphologically identified as a male. With regard to this latter observation, the occurrence of such histological disruption may have been underestimated because during sampling we targeted females and males on the basis of clear morphological differences; therefore, individuals which could not be ascribed to a sex were not sampled. Finally, in addition to these maturation defects, seminoma have been observed in PY-3X, HO-1X and LO-3X as previously reported [31].

Crosses performed between Control and exposed fish (HO-1X and LO-3X) suggest that spawning success is mainly driven by females, at least for the petrogenic fractions investigated. Beyond strictly reproductive defects, this could be due to behavioral disruptions which we have previously reported [32].

Overall, the present results showed that lifelong exposures to environmentally relevant PAHs fractions lead to reproductive dysfunctions and that these defects depended on the fraction used. Differences could be seen both on defects’ severity with petrogenic fractions producing more dramatic effects than PY fraction, and on defects nature with some specific insults such as ovaries degeneration after PY exposure or fertilization rate decrease after LO exposure. Such differences suggest the triggering of different molecular mechanisms.

### 4.2. Underlying Mechanisms

Reproduction defects can be the result of a general physiological dysfunction. In regard to this, it should be said that several physiological traits have been measured on the same fish and that, beyond the behavioral disruptions already mentioned, growth has been shown to be reduced after exposure, as well as the expression of some digestive enzyme and the ability to cope with food deprivation [33]. This reduced growth (along with disrupted condition indices) has already been observed upon exposure to PAHs [24,46,47,48] and could also have contributed to reduced reproduction output.

Exposure to PAHs usually results in a decrease in estradiol (E2) and 11-ketotestosterone (T), this has been observed after exposure to individual compounds [26,43,49] and more complex mixtures such as Produced water [44]. However, in other cases, an increase is observed [50] or E2 is reduced while T is increased [41]. In some cases, the situation is more complex with non-monotonic responses [27,45]. Finally, in some cases no change is observed after exposure to individual compounds or complex mixtures [16,51]. Altogether, these results suggest that PAHs can have endocrine disrupter activity while the estrogenic and/or anti-estrogenic property is not clear. In our cases, E2, T and vitellogenin circulating concentrations have been measured but no clear conclusion could be drawn (data not shown). In order to gain insight into molecular mechanisms underlying the observed reproduction defects, we have analyzed the expression of a set of genes involved in different functions (steroid pathways, gonad differentiation and maintenance) in gonads sampled at 9 mpf. Gene’s expression changes not follow a dose-response scheme, in particular very few changes were observed with the 3X concentration (not performed for HO since no more fish were available at this age likely resulting from an excessive toxicity). According to *esr* regulation by E2 [52], the activation in ovaries of *esr1* and *esr2a* expression in PY-1X and HO-0.3X and 1X and of *cyp19a1a* in PY-1X as well as the decrease in *esr2b* in LO-1X are indicative of an estrogenic activity of these fractions in females. Another explanation could be that AhR directly activates the expression of estrogen receptors [53,54]. The reduction of *cyp17a1* expression in testis of PY-0.3X and HO males is also indicative of estrogenic activity in males according to the known regulation of Cyp17a1 protein by E2 in zebrafish [55]. In testis, *spag8* is expressed in the acrosomal region of spermatozoa and in germ cells starting in spermatocytes [56] and its expression is positively regulated by androgen receptor agonists and negatively by antagonists [57]. The observed decrease in *spag8* expression may be indicative of anti-androgen activity in PY exposed fish. This combination of estrogenic and anti-androgenic activity may explain the gonadal degeneration in PY females’ ovaries; indeed both an excess of estrogen and a decrease in testosterone have been shown to promote ovarian structural disruption [58,59,60]. Besides, it has been shown in mouse that PAHs exposure promoted ovarian failure through AhR-mediated *bax* activation [61]. This pathway could underpin the specific disruption observed in PY ovaries since PY fraction contains high proportion of high molecular weight PAHs which are supposed to be AhR ligands. This mechanism could also explain why, while in HO and LO the decrease in the number of mature follicle is compensated by a higher proportion of pre-vitellogenic follicles, this is not the case in PY-3X ovaries. Indeed, it has also been shown that PAHs triggering AhR such as PY fraction (and HO to a lower extent) also specifically destroys primordial oocytes [62]. On the male side, the regulation of *amh* expression in testis is of particular interest. Indeed, in mammals, this gene is expressed during gonadal development and later almost exclusively in Sertoli cells during fetal and prepubertal stages. Later on, *amh* remains expressed but at a lower level in relation with spermatogenesis. The up-regulation of *amh* in testis of LO males could indicate a persistent defect in testis differentiation and/or spermatogenesis and may explain the specific and dramatic lower fertilization rates observed in pair-wise crosses between females Control and males LO-3X. Altogether, these results are evocative of endocrine disruption but also suggest that in some cases, reproductive dysfunction may be the consequence of gonad differentiation and/or maturation defects leading to a high proportion of immature individuals. In this context, the single ovo-testis observed in HO-3X may be seen as a result of an incomplete switch from ovary-like pre-differentiation gonad to testis rather than a sex-reversal. As in the case of reproductive traits, molecular analyses point out differences between fraction’s effects, and PY and HO appear to share common pathways likely triggered by AhR while LO fraction would be acting through a yet-unidentified pathway.

### 4.3. Environmental Relevance

Field monitoring as well as environmental assessment after an oil spill have demonstrated the important consequences of PAHs exposure on ecosystems in general and fish in particular [63,64,65]. Identified effects cover a wide range of physiological functions or processes and include reproduction or reproductive traits defects. In the case of an oil spill, the situation is simpler since PAHs source is clearly identified. However, oil denominations cover a large range of mixtures, the compositions of which depend on extraction localization as well as refining level. This is exemplified by the two petrogenic fractions used in this study. HO fraction contained 31% of high molecular weight PAHs while they represent only 6% of LO fraction but both contained high proportion of methylated PAHs (45%–59%) [33]. The situation is less simple in areas where PAHs sources are diverse, often resulting from human activity. In this case, PAHs can be of both pyrolytic and petrogenic origin and enter aquatic environments through the deposition of atmospheric emissions or from soil runoff. PY fraction extracted from sediment collected in the Seine River is representative of such PAHs mixture encountered in urbanized areas. PY fraction contained a very high proportion of high molecular weight PAHs (82%) and very few methylated derivatives (5%) [33]. Another major difference with oil spill is that, in the latter case, PAHs emission is constant, leading to chronic exposure. Nonetheless, after acute exposure during an oil spill, and despite cleaning operations, a secondary chronic exposure to lower level of PAHs persist over a long time period as this has been extensively studied after the Exxon Valdez spill [64] converging toward similar issues as in urbanized areas.

The concentration of PAHs in the biota therefore varies depending on the surrounding concentrations, in particular in sediment, and on a trophic level. For example, the total concentration of US-EPA 16 PAHs in the copepod *Eurytemora affinis* in the Seine Estuary can reach 3.9 µg·g^−1^ [66] and in mussels can be as high as 1.6 µg·g^−1^ [67]. In the case of acute accidental exposure, the concentration in mussels can be even higher, for example 3.0 µg·g^−1^ after the Erika oil spill [68] and above 14 µg·g^−1^ after Exxon Valdez oil spill [69]. PAHs concentrations in 1X diets used within the frame of this study are in the (4.7–6.7 µg.g−1 dw) range [33] and therefore fall into concentration encountered in contaminated areas. Fish can be exposed to PAHs through several routes including the trophic route, which has been shown to be an important route [9,10,11].

## 5. Conclusions

Field monitoring has revealed correlation between PAHs concentrations in the environment (or metabolites in individuals) whatever PAHs origin, and reproductive defects in fish [11,20,21,22,23,24,25,29,63,70,71]. This kind of monitoring analysis is essential for the environment but biomarkers often only allow raw estimates of disruptions and whatever actual reproduction output cannot be measured. Effects on population are therefore often only reached through modeling approaches [1,72]. Moreover, we believe that complementary approaches such as the one presented here, are suitable to investigate environmental exposure to complex mixtures and that, even if we conducted controlled experimental exposures, our results are pinpointing serious alteration of the recruitment success in the wild fish population. Further characterizations of the pathways are needed to refine and develop smarter biomarkers and we believe that zebrafish could provide a useful toolbox for this purpose as well as for transgenerational effects assessment since offspring insult has been shown upon exposure to PAHs [41,46,73].

## Figures and Tables

**Table 1:** Summary of group spawning monitoring.

Figures	PY ^1^				HO ^2^				LO ^3^			
	Control	0.3X	1X	3X	Control	0.3X	1X	3X	Control	0.3X	1X	3X
Males	19	31	36	43	46	39	47	ND	31	55	25	27
Females	63	39	44	38	25	30	24	ND	37	29	37	25
Assays	87	87	87	87	37	37	37	19	53	53	53	53
Spawns	60	**43**	**35**	**1**	19	17	**0**	**0**	41	**29**	**25**	**0**
Eggs (total)	6880	2921	4836	48	1065	582	0	0	6254	2498	1307	0
Eggs (fert.)	5144	1927	3673	37	476	582	0	0	4894	1656	670	0
Fert. rate (%)	74.8	66.0	76.0	77.1	88.1	81.8	-	-	78.3	66.3	51.3	-

ND, not determined because of the small size of those fish. Spawns row indicates the number of successful spawns. Numbers in bold indicate success rates significantly different from Control group (*p* < 0.05); ^1^ PY, pyrolytic; ^2^ HO, heavy oil; ^3^ LO, Light crude oil.

**Table 2:** qPCR genes expression analysis in ovary of exposed fish.

**PY**	**0.3X**		**1X**		**3X**	
	**Fold Change**	**95% CI**	**Fold Change**	**95% CI**	**Fold Change**	**95% CI**
*esr1*	0.866	0.467–1.523	**1.871**	**0.738–5.330**	1.52	0.471–6.460
*esr2a*	1.225	0.329–3.330	1.047	0.254–3.257	0.798	0.214–2.638
*esr2b*	0.906	0.329–2.009	1.24	0.531–2.990	1.028	0.269–4.046
*cyp19a1a*	1.265	0.373–5.167	**2.142**	**0.632–7.352**	1.094	0.287–4.970
**HO**	**0.3X**		**1X**		**3X**	
	**Fold Change**	**95% CI**	**Fold Change**	**95% CI**	**Fold Change**	**95% CI**
*esr1*	**3.133**	**1.517–9.697**	1.257	0.488–4.096	Not done	-
*esr2a*	**5.029**	**3.323–9.452**	**3.043**	**1.182–5.624**	Not done	
*esr2b*	Not done		Not done		Not done	
*cyp19a1a*	1.666	0.573–3.221	1.265	0.686–3.414	Not done	
**LO**	**0.3X**		**1X**		**3X**	
	**Fold Change**	**95% CI**	**Fold Change**	**95% CI**	**Fold Change**	**95% CI**
*esr1*	0.89	0.339–2.869	0.599	0.182–2.501	0.642	0.220–2.272
*esr2a*	0.847	0.120–8.124	0.951	0.354–4.418	1.26	0.378–8.276
*esr2b*	0.747	0.315–2.530	**0.455**	**0.125–1.870**	0.486	0.185–1.661
*cyp19a1a*	1.013	0.041–5.064	0.483	0.054–3.590	**0.336**	**0.061–1.206**

**Table 3:** qPCR genes expression analysis in testis of exposed fish.

**PY**	**0.3X**		**1X**		**3X**	
	**Fold Change**	**95% CI**	**Fold Change**	**95% CI**	**Fold Change**	**95% CI**
*amh*	0.777	0.484–1.189	0.594	0.439–0.803	1.422	0.729–3.459
*spag8*	**0.449**	**0.280–0.776**	**0.509**	**0.382–0.677**	0.751	0.483–1.109
*cyp17a1*	0.528	0.207–1.623	**0.447**	**0.329–0.609**	0.948	0.385–1.948
*hsd11b3*	0.632	0.337–1.241	0.885	0.552–1.437	0.763	0.472–1.334
**HO**	**0.3X**		**1X**		**3X**	
	**Fold Change**	**95% CI**	**Fold Change**	**95% CI**	**Fold Change**	**-**
*amh*	0.885	0.614–1.534	0.739	0.191–1.600	Not done	-
*spag8*	1.496	0.764–2.857	1.381	0.600–3.074	Not done	
*cyp17a1*	**0.23**	**0.091–0.708**	**0.354**	**0.208–0.750**	Not done	
*hsd11b3*	0.917	0.578–1.644	0.93	0.603–1.611	Not done	
**LO**	**0.3X**		**1X**		**3X**	
	**Fold Change**	**95% CI**	**Fold Change**	**95% CI**	**Fold Change**	**95% CI**
*amh*	**2.109**	**1.031–3.978**	**1.723**	**1.369–2.061**	1.864	1.173–3.549
*spag8*	1.127	0.800–1.653	1.078	0.995–1.178	0.667	0.311–1.074
*cyp17a1*	0.977	0.470–2.123	**1.39**	**1.008–1.971**	1.104	0.574–2.174
*hsd11b3*	1.362	0.700–2.230	1.331	0.943–1.995	0.918	0.541–1.530

## Supplementary Materials

The following are available online at www.mdpi.com/2305-6304/4/4/26/s1. Table S1: Detailed concentration of individual PAHs in produced diets.
