# Supplementary material for: Fish Reproduction Is Disrupted upon Lifelong Exposure to Environmental PAHs Fractions Revealing Different Modes of Action
Source: Toxics. 2016 Oct 28;4(4):26. doi: 10.3390/toxics4040026 (PMC5606653; doi:10.3390/toxics4040026)
Supplement: Supplementary file 1 [file toxics-04-00026-s001.pdf]

Supplementary Materials: Fish Reproduction Is Disrupted upon Lifelong Exposure to Environmental PAHs Fractions Revealing Different Modes of Action

Caroline Vignet, Thibaut Larcher, Blandine Davail, Lucette Joassard, Karyn Le Menach, Tiphaine Guionnet, Laura Lyphout, Mireille Ledevin, Manon Goubeau, Hélène Budzinski, Marie-Laure Bégout and Xavier Cousin

Table S1. Detailed concentration of individual PAHs in produced diets (ng·g^{-1} food; mean ± SD; *n* = 4–7).

		PY				HO				LO			
	Ring #	Control	0.3X	1X	3X	Control	0.3X	1X	3X	Control	0.3X	1X	3X
naphthalene	2	6 ± 6	15 ± 7	56 ± 14	157 ± 74	4 ± 1	37 ± 6	120 ± 16	405 ± 73	3 ± 1	161 ± 21	315 ± 183	1110 ± 472
acenaphthylene	2	1 ± 0	11 ± 2	35 ± 6	114 ± 23	1 ± 0	3 ± 1	3 ± 1	13 ± 13	1 ± 0	15 ± 1	46 ± 2	136 ± 19
acenaphthene	2	2 ± 1	14 ± 12	29 ± 7	89 ± 24	27 ± 26	46 ± 23	74 ± 19	190 ± 17	23 ± 21	37 ± 26	67 ± 68	90 ± 30
fluorene	2	2 ± 1	14 ± 3	42 ± 8	137 ± 28	2 ± 1	34 ± 2	99 ± 8	312 ± 25	2 ± 1	79 ± 1	232 ± 2	677 ± 55
dibenzo[*b*,*d*]thiophene	2	2 ± 3	11 ± 1	34 ± 5	102 ± 26	0 ± 0	54 ± 3	166 ± 11	546 ± 54	0 ± 0	443 ± 8	1161 ± 18	3489 ± 208
phenanthrene	3	8 ± 4	95 ± 21	291 ± 60	895 ± 213	6 ± 3	152 ± 7	418 ± 32	1279 ± 51	6 ± 4	178 ± 7	492 ± 10	1438 ± 86
anthracene	3	1 ± 0	49 ± 13	159 ± 41	482 ± 165	0 ± 0	22 ± 1	70 ± 2	220 ± 10	0 ± 0	2 ± 1	4 ± 1	42 ± 54
fluoranthene	3	3 ± 3	130 ± 28	523 ± 182	1782 ± 353	2 ± 1	17 ± 1	44 ± 2	145 ± 18	2 ± 1	2 ± 2	1 ± 1	15 ± 17
pyrene	4	3 ± 3	112 ± 24	447 ± 160	1496 ± 311	1 ± 0	80 ± 3	227 ± 19	709 ± 64	2 ± 0	13 ± 2	18 ± 2	73 ± 28
benzo[a]anthracene	4	1 ± 0	171 ± 75	581 ± 221	1671 ± 763	0	57 ± 4	172 ± 11	543 ± 29	0	4 ± 1	14 ± 2	49 ± 28
triphenylene + chrysene	4	1 ± 0	215 ± 91	744 ± 290	2144 ± 1032	ND	108 ± 8	336 ± 21	1073 ± 79	ND	30 ± 1	98 ± 4	320 ± 62
benzo[*b*]naphto[2,1-*d*]thiophene	4	5 ± 3	52 ± 26	156 ± 70	472 ± 230	0	56 ± 4	186 ± 8	573 ± 36	0	66 ± 1	194 ± 9	588 ± 30
benzo[b]fluoranthene+benzo[k]fluoranthene+benzo[j]fluoranthene	4	2 ± 1	273 ± 72	868 ± 220	2740 ± 674	2.4	32 ± 2	110 ± 8	363 ± 18	2.4	6 ± 0	20 ± 1	66 ± 7
benzo[*e*]pyrene	5	1 ± 0	109 ± 29	346 ± 86	1084 ± 286	ND	56 ± 3	173 ± 11	536 ± 26	ND	17 ± 0	52 ± 1	160 ± 10
benzo[a]pyrene	5	1 ± 0	118 ± 33	373 ± 95	1168 ± 346	0 ± 0	ND	108 ± 6	342 ± 10	0 ± 0	ND	4 ± 1	17 ± 3
perylene	5	1 ± 0	37 ± 9	121 ± 27	390 ± 83	0 ± 0	ND	56 ± 4	172 ± 14	0 ± 0	ND	5 ± 1	13 ± 1
indeno(1,2,3-cd)pyrene	5	0 ± 0	123 ± 41	349 ± 89	1188 ± 265	ND	ND	ND	ND	ND	ND	ND	ND
dibenz(ah)anthracene + dibenz(ac)anthracene	5	2 ± 2	32 ± 11	108 ± 43	301 ± 106	0.3	ND	34 ± 2	113 ± 6	0.3	ND	3 ± 1	11 ± 1
benzo[ghi]perylene	6	0 ± 0	87 ± 27	268 ± 67	893 ± 191	0 ± 0	ND	146 ± 10	481 ± 12	0 ± 0	ND	12 ± 0	42 ± 12
**Sum PAHs**		**34 ± 6**	**1670 ± 448**	**5532 ± 1383**	**17305 ± 4798**	**39 ± 30**	**880 ± 28**	**2558 ± 169**	**8082 ± 305**	**33 ± 26**	**1053 ± 63**	**2739 ± 231**	**8335 ± 854**
2-methylnaphthalene	2	8 ± 9	15 ± 3	43 ± 8	116 ± 39	4 ± 1	137 ± 13	396 ± 32	1259 ± 118	4 ± 1	420 ± 19	1036 ± 96	2982 ± 725
1-methylnaphthalene	2	4 ± 6	8 ± 2	22 ± 5	62 ± 21	2 ± 0	86 ± 8	261 ± 20	854 ± 84	2 ± 0	458 ± 19	1147 ± 102	3300 ± 739
**Sum methylnaphthalenes**		**12 ± 15**	**23 ± 5**	**65 ± 13**	**178 ± 60**	**6 ± 2**	**223 ± 20**	**657 ± 52**	**2113 ± 201**	**5 ± 1**	**878 ± 38**	**2183 ± 197**	**6282 ± 1465**
3-methylphenanthrene	3	2 ± 1	17 ± 3	47 ± 11	149 ± 31	1 ± 0	126 ± 24	326 ± 47	934 ± 160	1 ± 0	114 ± 14	319 ± 38	850 ± 156
2-methylphenanthrene	3	2 ± 0	19 ± 5	60 ± 13	175 ± 41	1 ± 0	149 ± 11	400 ± 28	1172 ± 166	1 ± 0	121 ± 2	340 ± 16	915 ± 124
2-methylanthracene	3	0 ± 0	8 ± 2	25 ± 6	78 ± 18	ND	ND	ND	409 ± 32	ND	ND	ND	32 ± 8
9-methylphenanthrene + 1-methylanthracene	3	1 ± 0	14 ± 4	49 ± 12	165 ± 56	1 ± 0	137 ± 20	360 ± 85	990 ± 206	1 ± 0	283 ± 29	798 ± 42	2226 ± 394
1-methylphenanthrene	3	1 ± 0	12 ± 3	37 ± 11	100 ± 28	0 ± 0	73 ± 8	227 ± 15	617 ± 71	1 ± 0	122 ± 4	348 ± 14	956 ± 97
**Sum methylphenanthrenes**		**7 ± 3**	**70 ± 15**	**218 ± 41**	**668 ± 157**	**2 ± 2**	**530 ± 34**	**1448 ± 144**	**4122 ± 581**	**2 ± 2**	**640 ± 35**	**1804 ± 67**	**4957 ± 731**
**Total PAHs**		**55 ± 12**	**1763 ± 468**	**5816 ± 1433**	**18151 ± 4983**	**47 ± 33**	**1633 ± 71**	**4663 ± 360**	**14317 ± 813**	**40 ± 29**	**2572 ± 96**	**6726 ± 278**	**19574 ± 1945**

Ring #: number of aromatic rings; ND: not detectable.
